# A systematic review of adult animal models investigating ECMO use for ARDS: where to from here

**DOI:** 10.1186/s40635-025-00781-5

**Published:** 2025-07-18

**Authors:** Muhtadi Alnababteh, Xizhong Cui, Mark Jeakle, Yan Li, Nancy Terry, Tom Gamble, Junfeng Sun, Shreya Kanth, Peter Q. Eichacker, Parizad Torabi-Parizi

**Affiliations:** 1 Critical Care Medicine Department, Clinical Center, National Institutes of Health, Building 10, Room 2C145, 9000 Rockville Pike, Bethesda, MD 20892 USA; 2 NIH Library, Office of Research Services, National Institutes of Health, Bethesda, MD 20892 USA; 3 National Heart, Lung, and Blood Institute, National Institutes of Health, Bethesda, MD 20892 USA

**Keywords:** ECMO, ARDS, Lung injury, Animal, Preclinical, Systematic review

## Abstract

**Background:**

Controlled clinical trials investigating ongoing questions about extracorporeal membrane oxygenation (ECMO) for patients with the acute respiratory distress syndrome (ARDS), including what the optimal mechanical ventilation (MV) tidal volume (TV) strategies are and whether ECMO potentiates injurious host responses, are difficult. We therefore conducted a systematic literature search and review to characterize studies investigating ECMO in adult animal lung injury models and to determine whether they inform these questions.

**Methods:**

A systematic literature search with relevant search terms was conducted of four data bases through 2/2/24.

**Results:**

Forty-five studies met inclusion criteria, and most parameters examined were represented similarly in studies with (*n =* 24) or without (*n =* 21) severe ARDS PaO_2_/FiO_2_s levels (≤ 100 mmHg or > 100 mmHg). Overall, while only 11 studies were published from 1971 to 2005, 5, 8, and 11 were published in subsequent 5-year periods up to 2020 and then 10 through 2/2/24 (Figure 1). Most studies investigated pig or sheep models (*n =* 32), but since 2016, six studies employed rat models. Eighteen studies administered lung lavage alone or with another lung injury challenge (17 with PaO_2_/FiO_2_s ≤ 100) and 9 used oleic acid. Although seven studies administered lipopolysaccharide, very different from clinical ARDS only one used a bacterial and none a viral challenge. Thirty-two studies employed V-V ECMO. The most frequent duration of ECMO investigated was 24 h in 16 studies but only 2 studies investigated longer periods (48 and 96 h). Differences in study questions, methodologies and outcome measures precluded formal meta-analysis. However, overall in studies that compared mechanical ventilation alone (MV) to ECMO groups or that compared differing ECMO groups: in 5 studies ECMO supported tidal volume reductions that approached apneic levels in 2; all but 1 of 10 studies indicated that ECMO with or without TV reductions either did not increase or reduced lung injury measures; 2 studies did while 4 did not find that ECMO aggravated molecular or cellular markers of inflammation; and only 2 studies examined host thrombotic responses with ECMO.

**Fig. 1:**
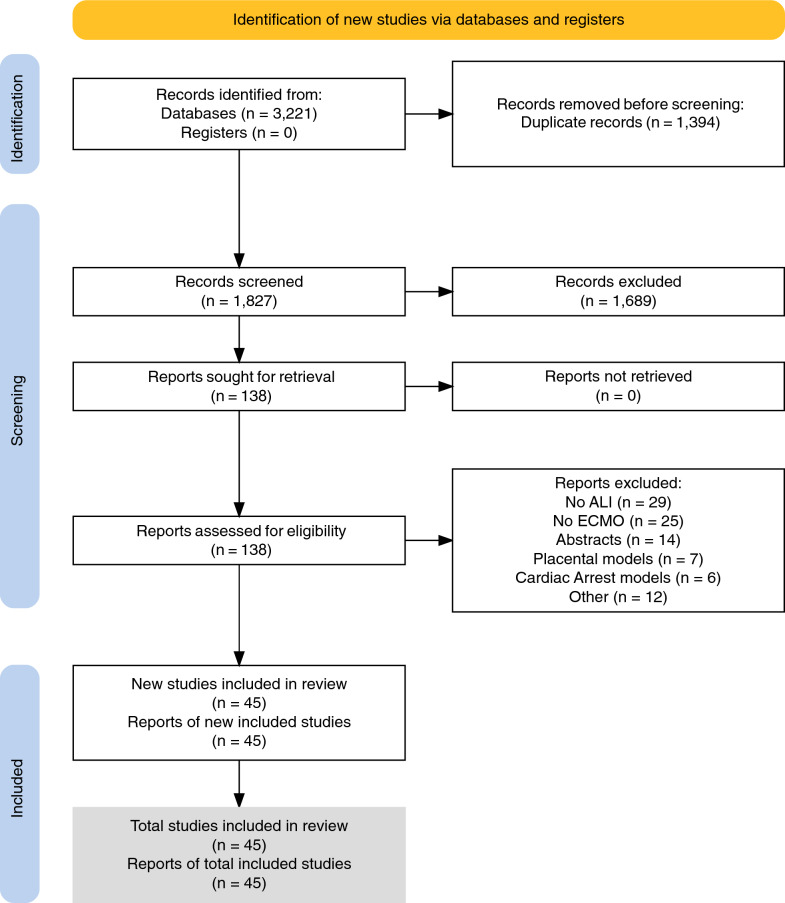
Flow diagram for the literature search

**Conclusion:**

Animal models to date have addressed important questions facing ECMO use for ARDS, but ones more closely simulating ARDS in patients appear warranted.

**Supplementary Information:**

The online version contains supplementary material available at 10.1186/s40635-025-00781-5.

## Background

Extracorporeal membrane oxygenation (ECMO) is increasingly employed in adults with progressive but potentially reversible acute respiratory distress syndrome (ARDS) [1–3]. Veno-venous ECMO (V-V ECMO), the most common form, as well as veno-arterial (V-AECMO) and arterial-venous ECMO (A-V ECMO), provide oxygenation and ventilatory support and may allow reductions in the potentially injurious higher tidal volumes and airway pressures related to mechanical ventilation alone [4, 5]. ECMO is most frequently applied for infection-related ARDS, and was used extensively during the SARS-CoV-2 pandemic for periods ranging from several days to weeks in COVID-19 patients [6–9]. Although its clinical application is increasing, questions remain about ECMO use [10–12]. While ECMO can support gas exchange, the best practices for mechanical ventilation (MV) such as tidal volume management are unclear [13–15]. There are also concerns that complications related to ECMO instrumentation, such as stimulated host immune and thrombotic responses, might aggravate underlying lung injury [10, 16–19]. Research is ongoing directed at these and other questions.

Animal models of ARDS and acute lung injury (ALI) contributed to the development of ECMO support for respiratory failure and continue to be used to understand and improve ECMO application [20, 21]. Preclinical ECMO research for ALI is evolving. For example, while a prior systematic review included only large animal models, several recent studies have investigated ECMO with ALI in rat models [20–22].

Therefore, to inform preclinical ECMO research going forward, we conducted a more current systematic review of studies investigating ECMO in adult animal models of ARDS and ALI. Because ECMO is typically administered to patients diagnosed with severe ARDS, retrieved studies were grouped for review based on whether the model of lung injury employed produced PaO_2_/FiO_2_ levels ≤ 100 or > 100. We first examined characteristics of the overall group of retrieved studies and categorized studies based on the design and types of comparisons made within studies. We then reviewed the questions asked, measures conducted, and conclusions made from each study. Our primary goal was to examine the research presently available from preclinical studies addressing with controlled study designs, the questions noted above regarding management of mechanical ventilation with ECMO and ECMO’s effects on lung injury and host inflammatory or thrombotic responses.

## Methods

This systematic review was prepared using the Preferred Reporting Items for Systematic Reviews and Meta-Analyses statement on guidance for literature review and data extraction [Supplemental File-A (SupFile-A)]. It was registered with the International Prospective Register of Systematic Reviews on December 20, 2022 (PROSPERO-2022-CRD42023773983).

### Literature search and study inclusion

Using search terms and strategies listed in SupFile-B, three authors (M.A., P.Q.E., P.T.P.) identified relevant studies published in the following databases from inception through February 2, 2024: PubMed, EMBASE, Web of Science and Biosis. These terms were modified from a previously published search of Pubmed and EMBASE [21]. Recovered reports were reviewed for additional references. Published studies that investigated either a veno-venous (V-V), veno-arterial (V-A) or arterio-venous (A-V) ECMO system established percutaneously in adult animals administered a challenge that would produce direct or indirect lung injury, were selected for further review. Non-English publications were excluded.

### Data extraction and organization of studies

Two investigators (M.A., P.Q.E) independently extracted data from reports using tables with formats comparable to those presented in this report. These data included: country and year of publication; species, strain, age and weight of animals; type, dose, route, timing and targeted level of the lung injury challenge or the level of hypoxemia resulting from the challenge; ECMO system, mode, duration, equipment, settings, ventilatory support, and anticoagulation employed; type and method of mechanical ventilatory support provided; and type of anesthetic agents and hemodynamic support used. Also extracted were the experimental groups compared, questions addressed, measures obtained, and a brief summary of the authors’ conclusions.

Studies were organized into two groups based on whether the level of lung injury targeted or produced resulted in severe ARDS PaO_2_/FiO_2_ levels (≤ 100 mmHg) or not (> 100 mmHg). Studies were further categorized based on the groups that were investigated and compared including: a mechanical ventilation alone (MV) versus a single ECMO group; an MV versus two different ECMO groups; two or more ECMO groups, all with lung injury; a single group with serial measures; or two ECMO groups, one with and one without lung injury.

To identify those studies employing concurrent controlled designs, either ECMO vs. MV groups or two of more ECMO groups, that investigated questions described in the introduction, the following criteria were employed. Studies investigating MV strategies for ECMO compared the effects of differing TVs, airway pressures (e.g., positive end expiratory pressures) or other MV maneuvers in MV vs ECMO groups or ECMO vs ECMO groups. Studies investigating the effects of ECMO on lung injury compared histologic lung injury measures (e.g., lung injury scores), wet-to-dry lung weight, or lung water measures in MV vs. ECMO groups. Studies investigating the effects of ECMO on host inflammatory or thrombotic responses compared inflammatory cytokines, other molecular markers of inflammation or inflammatory cell populations or measures of coagulation and thrombosis in MV vs. ECMO groups.

### Data synthesis and analysis

Relevant study information was summarized and presented in figures and tables where appropriate. Based on differences in the models and methods employed and endpoints investigated across studies, meta-analysis was not included in this systematic review.

## Results

The literature search retrieved 3221 reports. After exclusion of 1394 duplicates and 1689 others based on review of titles and abstract, 138 reports underwent full paper review, of which 45 reports were found to meet inclusion criteria [15–17, 22–63] (Supplemental [21]) Figure 1 (Sup Fig. S1).

### Study characteristics

Study characteristics are summarized in Table 1A and B for studies grouped based on PaO_2_/FiO_2_ levels targeted or reported and the groups compared as described in the methods. The overall number of included reports steadily increased over time, with 11 published from 1971 to 2005, but then 5, 8, and 11 published in each subsequent 5-year period up to 2020 and 10 through 2/2/24 (Figure 1). Whether studies targeted or reported PaO_2_/FiO_2_ levels ≤ 100 mmHg (*n =* 24 studies) vs. > 100 mmHg (*n =* 21 studies) was variable over the different time periods. While most studies investigated either pig or sheep large animal models (*n =* 18 and 14, respectively), since 2016, 6 studies employed rat models including one with PaO_2_/FiO_2_ levels ≤ 100 mmHg. Most studies (*n =* 31) included between 11 and 30 animals. No study examining PaO_2_/FiO_2_ levels ≤ 100 mmHg employed > 30 animals.

**Fig. 2:**
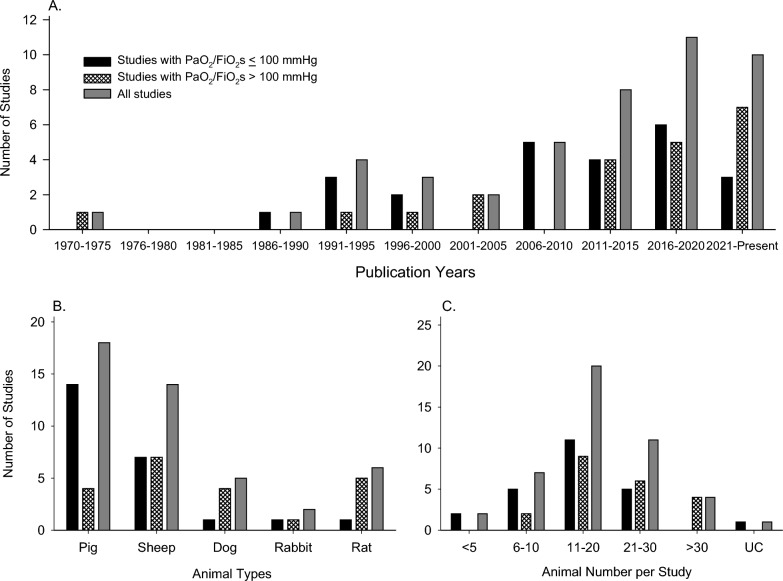
This figure shows the number of studies with PaO_2_/FiO_2_s ≤ 100 mmHg or PaO_2_/FiO_2_s > 100 mmHg and overall examining extracorporeal membrane oxygenation (ECMO) in adult animal lung injury models included in this systematic review that; were published during five year periods beginning in 1970 (**A**); employed either pig, sheep, dog, rabbit or rat models (**B**); and employed either ≤ 5, 6 to 10, 11 to 20, 21 to 30, > 30 or an unclear number of animals (**C**)

**Fig. 3:**
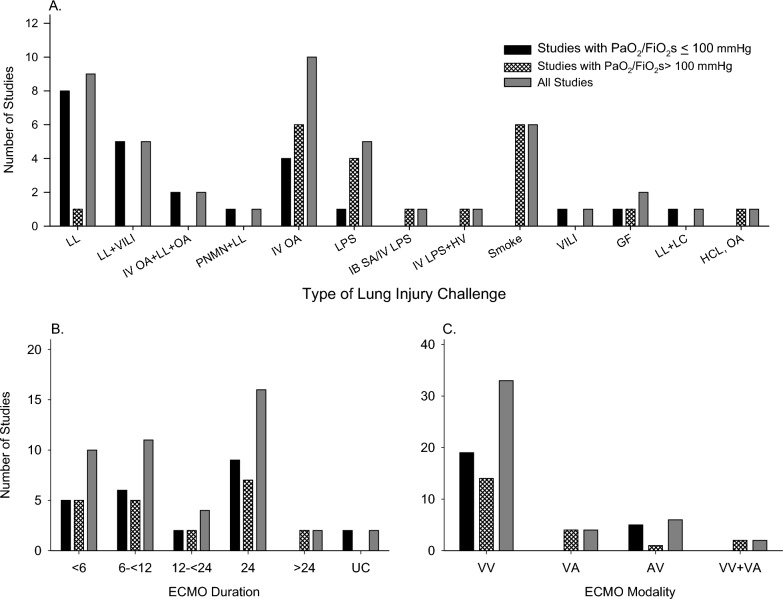
This figure shows the number of studies with PaO_2_/FiO_2_s ≤ 100 mmHg or PaO_2_/FiO_2_s > 100 mmHg and overall examining extracorporeal membrane oxygenation (ECMO) in adult animal acute lung injury models and included in this systematic review that; employed the different types of lung injury challenges reported on (**A**); investigated either veno-venous (V-V), veno-arterial (V-A), arterial-venous (AV), or both V-V and V-A ECMO systems; and investigated ECMO over < 6 h, 6 to < 12 h, 12 to < 24 h, 24 h, > 24 h or for an unclear period (**C**)

Over all studies, lung lavage alone and intravenous oleic acid alone were the most common types of lung injury challenges employed (9 and 11, respectively) but lung lavage was also used in combination with other challenges in 9 studies (Table 1, Fig. 2). While seven studies employed a lipopolysaccharide (LPS) lung injury challenge, only one employed a bacterial challenge (intrabronchial *S. aureus*) and this was in combination with LPS. No study used a virus challenge. While 16 of the studies with PaO_2_/FiO_2_s ≤ 100 mmHg employed lung lavage alone or combined with another challenge, only one study with PaO_2_/FiO_2_s > 100 mmHg used this challenge type. About equal numbers of studies grouped by PaO_2_/FiO_2_ level employed oleic acid but the only study with a bacteria challenge, most with LPS challenges, and all with smoke challenges targeted or resulted in PaO_2_/FiO_2_s > 100 mmHg (see Fig. 3).

**Table 1:** A and B Summary of study characteristics

Author	Publication		ECMO		Lung injury type	Animal	
	Year	Country	Mode	Time (h)		Type	Total number
A. Studies with PaO_2_/FiO_2_ ≤ 100							
Studies that compared an MV alone group with an ECMO group							
Plotz	1993	Netherlands	V-V	7	LL	Rabbit	12
German	1996	Austria, US	V-V	6	IV OA	Sheep	30
Iglesias	2008	Spain	A-V	12	PNMN-LL	Pig	15
Araos	2016	Chile	V-V	24	LL + VILI	Pig	18
Huang	2022	China	V-V	4	IV OA	Rat	UC
Studies that compared an MV alone group to two different ECMO groups							
Yanos	1990	US	V-V	6	IV OA	Dog	18
Johannes	2014	Austria, Germany	A-V	24	LL	Sheep	24
Pilarczyk	2015	Germany	V-V	8	LL	Pig	14
Studies that compared differing ECMO groups all with lung injury							
Hirschl	1995	US	V-V	4	LL + IV OA	Sheep	12
Hirschl	1996	US	V-V	4	LL + IV OA	Sheep	10
Kopp	2010	Canada, Germany	V-V	24	LL	Pig	24
Kopp	2012	Canada, Germany	V-V	24	LL	Pig	24
Araos	2019	Chile	V-V	24	LL + VILI	Pig	24
Dubo	2020	Chile	V-V	24	LL + VILI	Pig	12
Millar	2020	Australia, UK, US	V-V	24	IB LPS	Sheep	14
Qaqish	2020	Canada	V-V	6	GF	Pig	9
Araos	2021	Chile	V-V	24	LL + VILI	Pig	20
Studies with a single group and serial measures							
Booke	1995	Germany	V-V	UC	VILI	Sheep	7
Brederlau	2006	Germany	A-V	2.7	LL	Pig	15
Zick	2006	Germany	A-V	UC	LL	Pigs	7
Muellenbach	2009	Germany	A-V	8	LL	Pig	8
Langer	2014	Italy, US	V-V	12/16	IV OA	Sheep	11
Mendes	2022	Brazil	V-V	5	LL + LC	Pig	5
Andresen	2018	Chile, China	V-V	24	LL + VILI	Pig	5

B Studies with studies with PaO_2_/FiO_2_ > 100							
Studies that compared an MV alone group with an ECMO group							
Zwischenberger	1993	US	V-A/V-V	96	Smoke	Sheep	29
Hayes	2015	Australia	V-V	2/24	Smoke	Sheep	43
MacDonald	2015	Australia	V-V	24	Smoke	Sheep	40
Du	2016	China	V-V	24	IP LPS	Rat	40
Passmore	2016	Australia	V-V	24	Smoke	Sheep	27
Passmore	2017	Australia	V-V	24	Smoke	Sheep	27
Stenlo	2021	Sweden, US	V-A	7	LPS	Pig	21
Kayumov	2022	Australia, Korea, SG	V-A	3	LPS	Rat	80
Lim	2020	South Korea	V-V	12	IB SA/IV LPS	Pig	8
Brusatori*	2023	Italy, Germany, UK, US	V-V	24	HCL, OA	Pig	24
Studies that compared an MV alone group to two different ECMO groups							
Zhang	2021	China	V-V	9	IV OA	Dog	30
Studies that compared differing ECMO groups all with lung injury							
Trittenwein	1999	Austria, US	V-V/ V-A	1	IV LPS + HV	Rabbit	18
Kim	2004	South Korea	V-A	2	IV OA	Dog	16
Prat	2015	France, Italy, US	V-V	10	IV OA	Sheep	9
Xing	2021	China	V-A	24	IT/IV LPS	Rat	15
Zhang	2022	China	V-V	2	IV OA	Rat	15
Study with differing ECMO groups with lung injury and also a single group with serial measures							
LeFrack†	1973	US	V-V	8	GF	Dog	14
Studies with a single group and serial measures							
Li	2021	China	V-V	3	IV OA	Rat	12
Ju	2018	China	A-V	48	IV OA	Dog	12
Studies that compared ECMO groups with and without lung injury							
*Dembinski*	*2003*	Germany	V-V	6	LL	Pig	12
Shekar	2015	Australia	V-V	12	Smoke	Sheep	20

A-V, arterio-venous; ECLA, extracorporeal lung assist; GF, gastric fluid; gps, groups; HCL, hydrochloric acid; HV, hypoventilation; IB, intrabronchial; IP, intraperitoneal; IT, intratracheal; IV, intravenous; LA, linoleic acid; LC, lung collapse; LL, lung lavage; OA, oleic acid; PNMN, pneumonectomy; Pub., publication; SG, Singapore; UK, United Kingdom; US, United States; V-V, veno-venous; SA, *S. aureus*; UC, unclear; VILI, ventilator induced lung injury

*Two experiments with different lung injury challenge types

^†^Two experiments each addressing a different comparison and questions

The ECMO modalities investigated included V-V, V-A or A-V ECMO in 32, 8, and 3 studies, respectively, and 2 examined both V-V and V-A systems. V-V ECMO was employed about equally in studies that targeted or achieved PaO_2_/FiO_2_ levels ≤ vs. > 100 mmHg. Over all studies, the most frequent duration of ECMO investigated was 24 h in 16 reports. These durations were also about equal comparing studies with PaO_2_/FiO_2_ levels ≤ vs. > 100 mmHg. However, two reports, both with PaO_2_/FiO_2_s > 100 mmHg, studied periods longer than 24 h (48 and 96 h) (see Fig. 3).

**Fig. 4:**
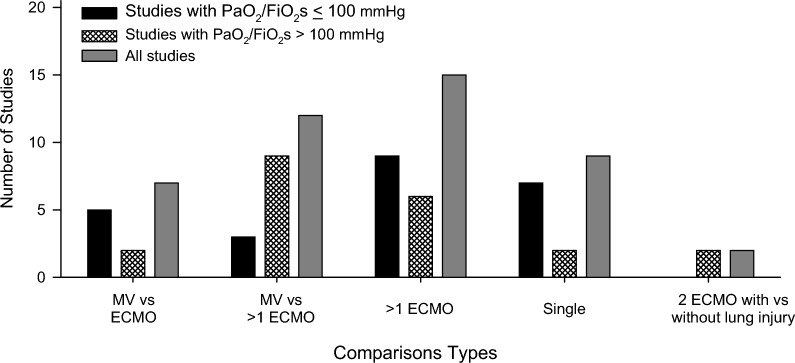
This figure shows the number of studies with PaO_2_/FiO_2_s ≤ 100 mmHg or PaO_2_/FiO_2_s > 100 mmHg and overall examining extracorporeal membrane oxygenation (ECMO) in adult animal lung injury models and included in this systematic review the numbers of studies that compared: a group with mechanical ventilation alone (MV) to a single group with ECMO (MV vs ECMO); an MV group alone to two ECMO groups (MV vs > 1 ECMO); more than one ECMO group (> 1 ECMO); a single group with serial measures (Single); and two ECMO groups, one group with and one group without lung injury (2 ECMO with vs without lung injury)

Studies made the following comparisons: 15 studies, 7 with PaO_2_/FiO_2_s ≤ 100, compared an MV alone group to an ECMO group; 4 studies, 3 with PaO_2_/FiO_2_s ≤ 100, compared an MV alone group to two ECMO groups; 14 studies, 9 with PaO_2_/FiO_2_s ≤ 100, compared 2 or more ECMO groups; 9 studies, 7 with PaO_2_/FiO_2_s ≤ 100, included single groups with serial measures; and 2 studies with PaO_2_/FiO_2_s > 100, compared 2 ECMO groups, 1 group with and 1 without lung injury (see Fig. 4). One study with PaO_2_/FiO_2_s > 100, included two experiments, one with a single group and serial measures and one with three ECMO groups [42]. Another study with PaO_2_/FiO_2_s > 100, also included two experiments, one employing a hydrochloric acid challenge and one using oleic acid [28]. Of note, seven of the studies comparing an MV group to an ECMO group with lung injury, all with PaO_2_/FiO_2_s > 100 mmHg, also included MV and ECMO groups that had not received lung injury challenges [16, 17, 30, 45, 49, 62, 63].

Additional details regarding lung injury challenges, anesthesia and hemodynamic support, and the ECMO employed in reviewed reports are provided in SupTables 1, 2, and 3. Of note, studies employing smoke challenges typically targeted carboxyhemoglobin and not oxygenation levels (SupTable 2). Because six rat models examining ECMO with lung injury were either not included in a prior review of ECMO in animal lung injury models (one study) or have been published since that review and because this type of small animal model may be of use by a wider number of investigator groups, we highlight methods in these studies in rats in Table 2.

**Table 2:** Summary of the lung injury, anesthesia and ECMO methodologies in studies employing rat models

Author	Year	Total N	Mode	Config	Time (h)	Lung injury type	Pump	Oxygenator	Blood Flow (mL/min)	Sweep Gas	Membrane Gas O₂%	Anesthesia (Ind.)	Anesthesia (Maint.)	ET	Comparisons
Du	2016	40	V-V	NA	24	IP LPS	NR	NR	NR	NR	NR	NR	Iso	NR	MV vs ECMO
Li	2021	12	V-V	RJV–RJV	3	IV OA	PreFluid Peristaltic	Xijing	80–90	80–100	0.90, Titrate to PaO₂	Sev	–	–	Single gp/serial measures
Xing	2021	15	V-A	RIJ–RCA	24	IT/IV LPS	Kewei	Kewei	150	200	1	Sumianxin, Zoletil	Sumianxin, Zoletil	ET	Differing ECMO gps
Huang	2022	UC	V-V	RJV–RJV	4	IV OA	PreFluid Peristaltic	Xijing	80–90	80–100	0.90, Titrate to PaO₂	Sevo	Sevo	ET	MV vs ECMO
Kayumov	2022	80	V-A	REJV–LFA	3	LPS	Watson-Marlow Pumps	Micro-1	50	NR	NR	Ket, Xylazine	Isoflurane	ET	MV vs ECMO
Zhang	2022	15	V-V	RJV–RJV	2	IV OA	PreFluid Peristaltic	Xijing	80–90	80–100	0.9	Pen	Pro or Sevo	ET	Differing ECMO gps

ALI, acute lung injury; ET, endotracheal tube; IP, intraperitoneal; IV, intravenous; IT, intratracheal; LPS lipopolysaccharide; OA, oleic acid; NR, not reported; PFP, PreFluid peristaltic pump; RCA, right carotid artery; REJV, right external jugular vein; RIJ, right internal jugular vein; RJV, right jugular vein; VA, veno-arterial; VV, veno-venous; Xijing, Kewei, Micro-1, commercial oxygenators; Iso, isoflurane; Sev, sevoflurane; Pen, pentobarbital; Pro, propofol; Ket, ketamine

### Study questions, measures and conclusions

Study questions, measures and conclusions are briefly summarized in Tables 3 and 4 for studies targeting or producing PaO_2_/FiO_2_ levels ≤ 100 mmHg and > 100 mmHg, respectively. In each table, studies are organized based on the types of comparisons made (see methods). Both groups of studies asked and reported a range of questions and conclusions, respectively. We were most interested in determining whether studies with concurrent controlled study designs had examined questions noted in the introduction regarding the management of MV with ECMO, and the effects of ECMO on lung injury and host inflammatory and thrombotic responses.

**Table 3:** Summary of study questions, measures and conclusions for studies with lung injury targeting or resulting in PaO_2_/FiO_2_s ≤ 100mmHg

Author (y)	Question	Measures*	Conclusions†
Studies that compared an MV alone group with an ECMO group^‡^			
Plotz (1993)	Compared surfactant with positive pressure-controlled ventilation vs. with V-V ECMO with sighs	Weaning success, GasEx^§^, lung mechanics^§^, ventilator efficiency	Large volume surfactant instillation was possible with V-V ECMO and sighs
German (1996)	Compare effects of MV with NO vs with V-V ECMO on Hemos, GasEx and lung lymph drainage during	Hemos^§^, GasEx, lung lymph flow and protein concentration	NO reduced PVR and increased oxygenation and lung lymph flow; ECMO improved oxygenation more and removed CO_2_
Iglesias (2008)	Compare conventional MV with 10-12mL/kg or 6mL/kg vs A-V ECMO with near-static ventilation	Weaning success, Hemos, GasEx, lung mechanics, LIS, lung IL-6, IL-8, TNFα, surfactant	A-V ECMO and near-static ventilation improved outcomes versus conventional MV
Araos (2016)	Compare the effects of V-V ECMO to MV alone	Survival, Hemos, GasEx, lung mechanics, LIS, W/D	ECMO rescued an MV refractory lung injury model and will allow testing of other V-V ECMO interventions
Huang (2022)	Investigate if V-V ECMO is protective and if Hippo/YAP signaling contributes to that protection	Oxygenation, LIS, W/D, AT2 cells, BAL and lung tissue IL-6, IL-8 and TNFα, Hippo/YAP signaling markers	V-V ECMO stimulated Hippo/YAP signaling aided recovery of injured alveolar epithelium
Studies that compared an MV alone group to two different ECMO groups			
Yanos (1990)	Investigate whether V-V ECMO supported hypopnea would reduce lung edema	Hemos, GasEx, lung mechanics, W/D, lung H_2_O	V-V ECMO supported hypopnea decreased Paw and PAP and increased PaO_2_ but didn’t alter lung water and increased venous admixture
Johannes (2014)	Compare effects of reducing TV to 3mL/kg or apneic ventilation with A-V ECMO vs. MV with 6mL/kg alone	Hemos, GasEx, lung mechanics, regional LIS	A-V ECMO allowed TV decreases to 0mL and reduced upper lung hyperinflation but increased lower lung inflammation
Pilarczyk (2015)	Compare conventional V-V ECMO to a miniaturized oxygenator with rotary pump	Hemos, GasEx, CBC, LFTs, free Hb, pump characteristics	Miniaturized system supported Hemos and GasEx but increased hemolysis
Studies that compared differing ECMO groups			
Hirschl (1995)	Investigate if liquid vs gas ventilation improves lung function during support with V-V ECMO	Hemos, GasEx, lung mechanics, histology	Liquid ventilation improved GasEx, lung compliance, and lung injury and inflammation
Hirschl (1996)	Investigate if total followed by partial liquid ventilation vs. gas ventilation improves lung function supported with V-V ECMO	Hemos, GasEx, lung mechanics, histology	Total followed by partial liquid ventilation improved GasEx, lung injury and inflammation but only total liquid ventilation improved compliance
Kopp (2009)	Investigate if a low resistance oxygenator without blood pump or an oxygenator with a miniaturized pump improves hemocompatibility vs. conventional V-V ECMO	Coagulation studies (ACT, PTT, PT, TAT, Fibrinogen, PLTs), thromboelastography, CBC, serum IL-8 and TNF, EM of filters	Both systems were hemocompatible but absence of a blood pump did not increase this
Kopp (2012)	Compare Hemos and GasEx with miniaturized V-V ECMO vs. A-V ECMO system	Hemos, GasEx and pump characteristics	Both systems facilitated LPV but A-V ECMO limited oxygenation and CO_2_ exchange and increased cardiac work
Araos (2019)	Compare nonprotective (10mL/kg), protective (6mL/kg) or near-apneic MV with V-V ECMO	Hemos, GasEx, lung mechanics, LIS, W/D, myofibroblast protein markers	ECMO supported near-apneic MV and reduced lung injury on histology and myofibroblast marker expression
Dubo (2020)	Investigate if low spontaneous breathing efforts worsens lung injury vs. controlled near-apneic ventilation during V-V ECMO	Hemos, GasEx, lung mechanics, vasopressors, fluid balance, lactate, LIS, W/D, lung cytokines	Low intensity spontaneous breathing with high RRs and low TVs did not worsen lung injury versus near-apneic controlled ventilation
Millar (2020)	Investigate safety and efficacy of mesenchymal stromal cell (MSC) administration with V-V ECMO	Hemos, GasEx, lung mechanics, LIS, W/D, BAL protein, ECMO characteristics	MSC diminished oxygenator function and did not improve oxygenation but reduced lung injury and inflammation on histology
Qaqish (2020)	Establish lung injury with V-V ECMO model with and without surfactant	Hemos, GasEx, lung mechanics, LIS, W/D, plasma and BAL inflammatory cytokines, BAL bile acid	A lung injury with V-V ECMO model developed and surfactant was tolerated but did not alter function
Araos (2021)	Compare PEEP 0, 10 and 20 levels during near-apneic ventilation with V-V ECMO	Hemos, GasEx, lung mechanics, LIS, W/D	V-V ECMO with PEEP = 10 limited lung injury, benefited GasEx, and did not worsen Hemos
Studies with a single group and serial measures			
Booke (1995)	Investigate if percutaneous V-V ECMO can provide lung support	Hemo, GasEx	V-V ECMO maintained gas exchange even in paralyzed animals after ALI
Brederlau (2006)	Investigate A-V ECLA’s contribution to gas exchange with different gas flows	Hemo, GasEx	A-V ECLA removed CO_2_ but oxygenation was reduced during severe hypoxia by increased shunt fraction
Zick (2006)	Test oxygenation with a pumpless interventional lung assist device (ILA)	GasEx, oxygenator blood flow	ILA significantly increased oxygenation but the effect was small
Muellenbach (2009)	Investigate TV reductions with A-V ECMO and an open lung approach	Hemos, GasEx, lung mechanics	A-V ECMO and an open lung approach provided CO_2_ removal with TVs of 0-2mL/kg and maintained oxygenation increases
Langer (2014)	Investigate V-V ECMO during spontaneous ventilation with 6 different gas flows, before and then after lung injury	GasEx, lung mechanics, lung CT scans, plasma IL-1b, TNF, IL-8, IL-10	V-V ECMO can control spontaneous ventilation in healthy sheep and ones with ARDS
Andresen (2018)	Investigate meropenem PKs and a rapid response PK biosensor during V-V ECMO supported	Meropenem PK measures	Biosensor provided reliable meropenem PK data which ECMO did not alter
Mendes (2022)	Investigate V-V ECMO effects on lung perfusion and Hemos during one-sided lung ventilation and lung collapse and lavage	Hemo, GasEx, lactate, Hb	ECMO decreased PAP and may have increased shunt, but did not alter lung perfusion distribution with varying V/Q mismatches

ACT, activated thromboplastin time; ALI, acute lung injury; AT2, alveolar type 2 cells; AV, arterio-venous; BAL, bronchoalveolar lavage; BUN, blood urea nitrogen; CBC, complete blood count; CO_2_, carbon dioxide; CT, computerized tomography; ECLA, extracorporeal lung assist; ECMO, extracorporeal membrane oxygenations; EM, electron micrography; FiO_2_, fractional inspired oxygen concentration; GasEx, gas exchange; GP, group; H_2_O, water; Hb, hemoglobin; Hemos, hemodynamics; IL, interleukin; LFTs, liver function tests; LIS, lung injury score on histology; LPV, lung protective ventilation; MV, mechanical ventilation; NO, inhaled nitric oxide; O_2_, oxygen; PaO_2_, arterial oxygen pressure; PAP, pulmonary artery pressure; Paw, airways pressure; PEEP, positive end expiratory pressure; PK, pharmacokinetics; PLT -platelet; PTT, partial thromboplastin time; PT, prothrombin time; PVR, pulmonary vascular resistance; Rx, treatment; TAT, thrombin–antithrombin complexes; TNF, tumor necrosis factor; TV, tidal volume; V-A, veno-arterial; V/Q, ventilation/perfusion; V-V, veno-venous; W/D, wet to dry lung ratio;

^*^Listed measures may not include all those reported in a study

^‡^†Summarized from reports’ findings or conclusions

^‡^ALI is employed whether a study designated the model as an ALI or acute respiratory distress syndrome one

^§^depending on the study hemodynamics included systemic and/or pulmonary vascular measures, gas exchange included measures of oxygenation and/or carbon dioxide removal and lung mechanics included static lung compliance and/or airway pressures

**Table 4:** Summary of study questions, measures and conclusions for studies with lung injury targeting or resulting in PaO_2_/FiO_2_s > 100mmHg

Author (y)	Question	Measures*	Conclusions^†^
Studies that compared an MV alone group to an ECMO group^‡^			
Zwischenberger (1993)	Investigate effects of S-ALI treated with V-A/V-V ECMO on blood inflammatory mediators and GasEx	Hemos^§^, GasEx^§^, CBC, plasma thromboxane B_2_, conjugated dienes, lung histology, W/D	ECMO exacerbated the inflammatory response to S-ALI and may have impaired GasEx
Hayes (2015)	Investigate V-V ECMO and hyperoxia effects on PLT dysfunction	Hemos, GasEx, ADP and collagen-induced PLT aggregation, Ca^2+^	ECMO did not alter PLT activity, but hyperoxia with ECMO may have desensitized ADP-dependent PLT activation
MacDonald (2015)	Investigate S-ALI, V-V ECMO and RBC Rx effects on oxidative stress and plasma selenium levels	Hemos, GasEx, LFTs, COHb%, plasma TBARS, GTX and selenium activity	S-ALI increased TBARS; Adding ECMO to S-ALI reduced selenium more than S-ALI and ECMO alone
Du (2016)	Investigate V-V ECMO effects during LPS induced lung injury	Hemos, GasEx, histology, W/D, lung stress kinases	ECMO aided oxygenation and cardiac function
Passmore (2016)	Investigate S-ALI and ECMO effects alone and together on lung and blood inflammatory cells, cytokines and tissue remodeling	GasEx, lung mechanics^§^, fluid balance, lung inflammatory cells, LIS, W/D, BAL protein and cells, plasma IL-1b, IL-6 and IL-8, HCT, lung MMP2 and 9	Addition of ECMO augments the inflammatory response in a host with pre-existing lung injury
Passmore (2017)	Investigate S-ALI and ECMO effects alone and together on parameters of hemostasis	CBC, coagulation and PLT function tests, pump flow	ECMO stimulated collagen-induced PLT aggregation and reduced factor VIII, vWF and fibrinogen levels, changes augmented with ECMO and lung injury together
Stenlo (2021)	Investigate whether a particle flow rate test (PFR) tracks lung injury during V-AECMO	Hemos, GasEx, lung mechanics, Hb, plasma and BAL IL-6, LIS, W/D, proteomics, PFR	A PFR test tracked lung injury
Kayumov (2022)	Investigated cardiac left ventricular performance and oxygen consumption with ECMO in LPS challenged rats	Left ventricular performance measures	ECMO in LPS challenged rats resulted in higher myocardial oxygen consumption
Lim (2020)	Compared ultralow 3mL/kg TVs with V-V ECMO to conventional MV with 15mL/kg TVs	Hemos, GasEx, BAL cytokines, lung ultrasound, LIS, W/D	V-V ECMO with low TVs was possible and protected against VILI
Brusatori (2023)	Compare V-V ECMO, ECCO_2_R and MV alone on Hemo, GasEx, lung mechanics with HCL or OA induced lung injury	Hemo, GasEx, lung mechanics, oxygen consumption	Compared to MV, ECMO had better effects on Hemo, GasEx and oxygen consumption than ECCO_2_R
Studies that compared an MV alone group to two different ECMO groups			
Zhang (2021)	Investigate whether CRRT reduces inflammatory markers during lung injury and V-V ECMO	Hemos, GasEx, Hb, plasma IL-6, TNFa (MV + ALI vs ECMO + ALI vs ECMO + Rx + ALI)	V-V ECMO and CRRT removed inflammatory mediators and improved oxygenation and hypercapnia
Studies that compared differing ECMO groups			
LeFrack (1973)^¶^	Establish a V-V ECMO supported gastric fluid lung injury model and compare Travenol, Peirce GE, Lande-Ed oxygenators	Hemos, GasEx, lung mechanics (Single gp/serial measures)	Travenol oxygenator delivered most O_2_ based on membrane surface area, Pierce GE had highest O_2_ transfer irrespective of area
Trittenwein (1999)	Compare if an oxygenator delivering FiO_2_ = 1.0 with V-V vs V-AECMO would increase lipid peroxidation and lung injury after LPS priming and hypoxia	Hemos, GasEx, lung tissue and plasma MDA levels	After hypoxia, ECMO with FiO_2_ = 1.0 in LPS challenged animals increased oxidative lipid damage, an effect dependent on PaO_2_ with V-V ECMO
Kim (2004)	Investigate whether a pressure relieving compliance chamber (PRCC) reduces RBC injury in a pulsatile V-AECMO system	Hemos, GasEx, plasma free Hb, CBC, LFTs, BUN	PRCC with a pulsatile pump decreased RBC trauma but not as well as a non-pulsatile pump alone; the pulsatile pump improved oxygenation
Prat (2015)	Compare one initial low dose of heparin vs a standard regimen, both with miniaturized V-V ECMO	CBC, coagulation factors, thromboelastography, PLT aggregometry and P-selectin, TAT, plasmin/antiplasmin complexes	Single low dose heparin bolus maintained anticoagulation for 10h
Xing (2021)	Investigate YTHDF1 KOmac effects on immunity during LPS challenge and V-AECMO	GasEx, inflammatory markers, flow cytometry, western blot, RNA methylation, brain histopathology	In V-AECMO supported septic rats, YTHDF1 KOmacs benefitted immune paralysis and brain injury
Zhang (2022)	Compare the effects of sevoflurane vs. propofol anesthesia with V-V ECMO on oxygenation and inflammatory injury	Hemos, GasEx; TNFa and IL-1b in BAL, serum and lung tissue; BAL protein, lung myeloperoxidase, LIS, W/D	Compared to propofol, sevoflurane with lung injury and ECMO improved oxygenation and reduced inflammatory markers
Studies with a single group and serial measures			
Li (2021)	Establish a rat model of V-V ECMO and lung injury	Hemos, GasEx, HCT, Hb, lytes, LIS, W/D, BAL protein	A V-V ECMO rat model of lung injury was established
Ju (2018)	Investigate correct by-pass flow for A-V ECLA and A-V ECLA’s effects on GasEx and inflammatory cytokines	Hemos, GasEx, serum cytokines, lactate and urine output	A-V ECLA provided GasEx without excessive inflammation or tissue hypoperfusion
Studies that compared ECMO groups with and without lung injury			
Dembinski (2003)	Investigate the safety and efficacy of V-V ECMO with heparin anticoagulation during normal and injured lung function	Hemos, GasEx, ECMO pump dynamics, CBC, fibrinogen, macroscopic examination of the pump and oxygenator for visible clots	During lung injury, V-V ECMO with low dose heparin provided stable Hemos, adequate GasEx, and no major blood damage or external clot formation
Shekar (2015)	Investigated whether several antibiotic properties predict antibiotic behavior with V-V ECMO	Hemos, GasEx, PK data for 8 different antibiotics	With ECMO, lipophilic antibiotics increased V_ss_ and decreased CL while protein bound antibiotics decreased V_ss_ and CL

ADP -adenosine diphosphate; ALI, acute lung injury; AV, arterio-venous; BAL, bronchoalveolar lavage; BUN, blood urea nitrogen; CBC, complete blood count; Cl, clearance; CRRT, continuous renal replacement therapy; ECCO_2_R, extracorporeal CO_2_ removal; ECLA, extracorporeal lung assist; ECMO, extracorporeal membrane oxygenations; FiO_2_, fractional inspired oxygen concentration; GasEx, gas exchange; GP, group; GTX, glutathione peroxidase; Hb, hemoglobin; HCL, hydrochloric acid; HCT, hematocrit; Hemos, hemodynamics; IL, interleukin; KOmac, knock out macs; LFTs, liver function tests; LIS, lung injury score on histology; LPS, lipopolysaccharide; Lytes, serum electrolytes; MDA, malondialdehyde; MMP, matrix metalloproteinase; MV, mechanical ventilation; O_2_, oxygen; OA, oleic acid; PaO_2_, arterial oxygen pressure; PK, pharmacokinetics; PLT -platelet; RBC, red blood cell; S-ALI, smoke induced ALI; TAT, thrombin–antithrombin complexes; TBAR, thiobarbituric; TNF, tumor necrosis factor; TV, tidal volume; V-A, veno-arterial; VILI, ventilator induced lung injury; V_ss_, steady state distribution volume; VV, veno-venous; vWF, von Willebrand factor; W/D, wet to dry lung ratio;

^*^Listed measures may not include all those reported in a study

^†^Summarized from reports’ findings or conclusions

^‡^ALI is employed whether a study designated the model as an ALI or acute respiratory distress syndrome one

^§^Depending on the study hemodynamics included systemic and/or pulmonary vascular measures, gas exchange included measures of oxygenation and/or carbon dioxide removal and lung mechanics included static lung compliance and/or airway pressures

^¶^This study included two sets of experiments, one with a single group and serial measures and another with differing ECMO groups all with ALI

In concurrent controlled studies comparing ECMO to MV groups, three with PaO_2_/FiO_2_s ≤ 100 mmHg examined the effects of lowering TVs with ECMO [35, 36, 58], while one study with PaO_2_/FiO_2_s > 100 mmHg study investigated this question [44]. ECMO supported TV reductions in these studies, although one reported increased lower lung inflammation with the maneuver [36]. In two studies by one group comparing different ECMO groups and with PaO_2_/FiO_2_s ≤ 100, ECMO supported reductions in TV in one [15] while one concluded that an intermediate PEEP level of 10 cmH_2_O would be optimal with ECMO [25]. Also, in studies comparing different ECMO groups with PaO_2_/FiO_2_s ≤ 100 mmHg, one reported large volume surfactant instillation was possible with ECMO [51] and two determined liquid vs. gas ventilation improved ECMO application, but these were both very short-term studies (7 h and 3 h, respectively) [33, 34].

Whether it was a primary question of study or not, four studies with PaO_2_/FiO_2_s ≤ 100 mmHg reported the effects of ECMO compared to MV alone on lung injury scores and/or lung wet/dry weight ratios or lung water measures [22, 24, 35, 58], while six studies with PaO_2_/FiO_2_s > 100 mmHg provided such data [17, 28, 30, 44, 55, 62] (Table 3). ECMO was either associated with no significant effects or with reductions in these measures in all but one study which reported increases in W/D after 96 h of ECMO [62]. One study with PaO_2_/FiO_2_s ≤ 100 mmHg which compared differing ECMO groups, reported that decreasing TV levels with ECMO produced progressive decreases in global LISs but not W/Ds [15]. Another study by this group which also compared differing ECMO groups but with differing PEEP levels, reported that PEEPs of 10 cmH_2_O reduced global LISs and PEEPs of 10 and 20 cmH_2_O reduced W/Ds [25]. In two studies comparing ECMO and MV groups, survival appeared improved in the ECMO vs. MV group [24, 28].

In studies comparing ECMO and MV groups, two with PaO_2_/FiO_2_s ≤ 100 [22, 35] and five with PaO_2_/FiO_2_s > 100 mmHg [17, 30, 44, 55, 59, 62] provided BAL, lung and/or plasma IL-1b, IL-6, IL-8 and/or TNFa levels or other molecular or cellular markers of inflammation. In six of these studies providing an overall assessment of ECMO’s effects, in four ECMO appeared to have beneficial ones [22, 35, 44, 45] and in two it was potentially harmful [17, 62]. Only two studies comparing ECMO and MV groups and both with PaO_2_/FiO_2_s > 100 mmHg, reported on the possible effects of ECMO on parameters of coagulation and thrombosis [16, 49]. In one, increased oxygen levels with ECMO may have desensitized ADP associated platelet activation [16] and in the other ECMO with smoke associated lung injury may have augmented platelet activation and reduced factor VIII, Von Willebrand factor and fibrinogen levels [49].

## Discussion

This systematic review of studies investigating ECMO in adult animal lung injury models included 45 reports, which was 28 more than the last systematic review in this area of research we are aware of [21]. While the increased number of overall studies in the present review may relate in part to our use of four rather than two databases, the pace of preclinical research examining ECMO for ALI is increasing. For example, different from the prior review which presented no rodent studies, the present one included six rat models, five of which were published in 2021 or later. Consistent with clinical ECMO use, most studies employed V-V ECMO. However, very different from what’s encountered clinically, only one study employed ECMO in an infectious disease model of pneumonia which was bacterial. Despite ECMO’s application in both the earlier H1N1 influenza and frequent use in the recent SARS-CoV-2 pandemics, no animal model investigated ECMO with a viral challenge. Also, very different from clinical use, only two studies employed ECMO for more than 24 h. Most studies either compared MV with one or more than one ECMO group (*n =* 19) or compared differing ECMO groups (*n =* 15) and these comparison types were similar between studies with PaO_2_/FiO_2_s ≤ 100 vs > 100 mmHg.

A notable difference between studies with models that targeted or produced PaO_2_/FiO_2_s ≤ 100 mmHg vs. > 100 mmHg was the type of lung injury challenge administered. Most of the former employed lung lavage alone or in combination with an additional challenge whereas most of the latter employed smoke or LPS challenges. About equal numbers of the two study types employed oleic acid challenges. These differences may reflect the potency of the different challenges. However, studies with smoke challenge typically targeted carboxyhemoglobin levels, which may not have produced the same level of injury and hypoxemia as either lung lavage or oleic acid. Also, LPS challenge sufficient to produce PaO_2_/FiO_2_s ≤ 100 mmHg may result in unacceptable hemodynamic instability, especially when used in combination with anesthesia.

While differences among study designs, models and endpoints prevented meta-analysis, limited numbers of controlled animal studies reviewed here did address questions facing the clinical application of ECMO. Several investigated and supported reductions in tidal volumes used with MV during ECMO and one examined how PEEP might be managed with ECMO [25]. Except for one 96 h study, ECMO did not appear to aggravate histologic and lung water measures and may, with or without TV reductions, have reduced injury in some studies [17, 23, 26, 30, 32, 37, 46, 57, 60, 64]. Similarly, markers of host inflammatory responses were not increased with ECMO in most but not all studies examining these [17, 23, 37, 46, 47, 64]. There were only two controlled studies examining how ECMO might alter potentially associated with thrombotic responses [29, 52].

Despite differing methodologies, the studies reviewed here all addressed questions important for the application of ECMO in the setting of lung injury. However, the limited duration of ECMO administration in these animal studies and the absence of infectious disease as a source of lung injury, limits their interpretation in the context of clinical ECMO use for ARDS. Longer periods of ECMO exposure in combination with either a bacterial or viral source of infection associated lung injury might provide further insights into the questions we examined in studies. Such studies would best combine strategies designed to limit potential injury related to accompanying MV, one goal of ECMO use clinically. Longer term models and the use of infectious challenges appear possible. Notably, one of the studies comparing ECMO and MV groups analyzed here, supported sheep for 96 h, a period approaching ones encountered in patients [62]. Also, a 96 h model of sedated and mechanically ventilated canines infected with *S. aureus* pneumonia has been employed extensively in the study of the treatment and support of sepsis [64–66]. However, such large animal models requiring continuous intensive care unit type support are resource intensive and not possible for most laboratories. But given the increasing clinical use of ECMO for ARDS and difficulty of conducting controlled ECMO clinical studies examining key questions like the optimal ventilatory management of ECMO patients and its net effects on lung injury measures, it may be appropriate for governmental health agencies to fund such research in longer term large animal models.

Our review does indicate that ECMO is being increasingly investigated in rat ALI models which are potentially less resource intensive than large animal ones [22, 30, 30, 43, 57, 63]. While the sensitivity of these rodent models to the depressive hemodynamic effects of sedation and analgesia may preclude longer term study, these models do provide the tools to examine ongoing questions such as the effect of ECMO systems on host inflammatory and thrombotic responses [67–69]. Rodent studies would be facilitated by the wider range of reagents available for biologic study in these species. However, among the studies cited here, there is considerable methodological heterogeneity in how models were constructed, including variation in circuit configuration (V–V vs. V–A), cannulation strategies, oxygenator design, flow parameters, and anesthetic regimens. These differences could shape important physiologic and immunologic responses, complicating cross-study interpretation and limiting reproducibility. Anesthetics employed such as isoflurane, ketamine/xylazine, or sevoflurane differ in their hemodynamic effects and influence on immune responses. Likewise, the most common lung injury models—lipopolysaccharide and oleic acid—activate differing inflammatory pathways and differ in both severity and relevance to human disease. Despite infection being the most frequent clinical trigger for ECMO, none of the included rat studies employed a live bacterial challenge. Future ECMO studies in rats should incorporate Gram-positive or Gram-negative bacterial models to better reflect clinical conditions and explore how pathogen-driven inflammation interacts with the extracorporeal circuit. Such bacterial pneumonia models have been widely used to investigate other aspects of inflammatory lung injury [70, 71]. The rat is also susceptible to strains of beta-coronavirus that can be studied in biosafety level-2 laboratories [72]. Since SARS-CoV-2 continues to cause clinical infection and has the potential to mutate into a more resistant form, studies of ECMO in rat beta-coronavirus models should be considered. The small size of the rat model allows study with larger numbers of groups and samples and would facilitate multifactorial study designs comparing the individual and combined effects of pulmonary infection and ECMO.

Several limitations of this study include the following. Non-English reports were not reviewed, but even without those, the study included 45 studies overall. None of the findings from studies comparing ECMO and MV groups or differing ECMO groups could be blinded ones. This is a similar problem that clinical trials contend with. This review did not include studies conducted in neonatal and infant lung injury models. Finally, as noted, the differences in the models employed, limited numbers of studies addressing questions now facing clinical application of ECMO for ARDS, and the lack of standardized reporting among studies precluded meta-analysis.

In conclusion, animal lung injury models made an important contribution to the development of ECMO to support patients with ARDS. Animal models will continue to be important for the development and refinement of ECMO system performance. Data from existing animal studies provide important insights into ongoing questions about the degree to which ECMO can support reductions in TVs and airways pressures and about the host’s inflammatory and thrombotic responses to ECMO. However, among animal studies going forward, ones addressing these questions in models more closely simulating common sources of lung injury and the durations of ECMO encountered clinically appear warranted.

## Supplementary Information

Additional file 1.

Additional file 2.

Additional file 3.

Additional file 4.

## Data Availability

Not applicable.
