# Supplementary material for: A systematic review of adult animal models investigating ECMO use for ARDS: where to from here
Source: Intensive Care Med Exp. 2025 Jul 18;13:74. doi: 10.1186/s40635-025-00781-5 (PMC12274183; doi:10.1186/s40635-025-00781-5)
Supplement: Supplementary file 2 — Additional file 2. [file 40635_2025_781_MOESM2_ESM.docx]

| **Sup-Table 1**. Anesthesia, sedation and hemodynamic support employed in studies | | | | | | | | | | |
| --- | --- | --- | --- | --- | --- | --- | --- | --- | --- | --- |
| Author (ref) | Pub. year | Anesthesia and Sedation | | | | | | Hemodynamic Support | | |
|  |  | Premed | Induction | Maintenance | Paralytic | Titration | Airway | Fluid | Vasopressor | Titration |
|  |  | **Studies with PaO_2_/FiO_2_≤100** | | | | | |  |  |  |
| ***Studies that compared an MV alone group with an ECMO group*** | | | | | | | | | | |
| Plotz | 1993 | NR | pen | pen | pan | Yes | trach | NR | NR | NR |
| German | 1996 | NR | thio | thio | NR | NR | NR | NR | NR | NR |
| Iglesias | 2008 | aza, thio | fen, pro | fen, pro | roc | NR | ET | NS | NE | NR |
| Araos | 2016 | ket, xyl | fen, mid | fen, ket, mid, | atra | yes | ET | NS | NE | NC/NR |
| Huang | 2022 | NR | sevo | sevo | NR | NR | ET | NR | NR | NR |
| ***Studies that compared an MV alone group to two different ECMO groups*** | | | | | | | | | | |
| Yanos | 1990 | NR | chlo, ure | NR | NR | Nr | ET | Dextran 6% in NS | NR | NR |
| Johannes | 2014 | NR | thio, fen | thio, fen | pan | NR | ET | NR | NR | NR |
| Pilarczyk | 2015 | NR | aza, ket, atro | pro, mid, fent | NR | Yes | ET | LR 3cc/kg/hr | NE | yes |
| ***Studies that compared differing ECMO groups*** | | | | | | | | | | |
| Hirschl | 1995 | NR | ket, gua | ket, gua | pan | yes | trach | NC | NC | NC |
| Hirschl | 1996 | NR | ket, gua | ket, gua | pan | yes | trach | NC | NC | NC |
| Kopp | 2010 | aza, ket | thio | thio, fen | NR | NR | ET | RL, HES | NR | NR |
| Kopp | 2012 | aza, ket | thio | thio, fent | NR | NR | ET | RL, HES | NR | NR |
| Araos | 2019 | ket, xyl | fen, mid | fen, ket, mid, | atra | yes | ET | NS | NE | NC/NR |
| Dubo | 2020 | ket, xyl | fen, mid | fen, ket, mid, | atra | yes | ET | NS | NE | NC/NR |
| Millar | 2020 | NR | ket, mida | Ket, mid, fen | vec | yes | ET | Sodium lactate | NE, vaso | yes |
| Qaqish | 2020 | NR | ket, mid, atro | pro, iso, remi | No | yes | trach | NS | none | NC |
| Araos | 2021 | ket, xyl | fen, mid | fen, ket, mid, | atra | yes | ET | NS | NE | NC/NR |
| ***Studies with a single group and serial measures*** | | | | | | | | | | |
| Booke | 1995 | NR | NR | pro, fen | pan | NC/NR | trach | NR | NR | NR? |
| Brederlau | 2006 | ket, xyla | thio | ket, mid, fen | vec | NR | ET | BE 500 mL to 2-6ml/kg/hr | NC | NC |
| Zick | 2006 | aza, atro | ket, pro, suf | suf, pro | NR | NR | ET | LR and 6% HES | NE | yes |
| Muellenbach | 2009 | ket | fen, thio | thio, fen | pan | NR | ET | BE 4-5cc/kg/hr  Colloid 500 mL | NR | NR |
| Langer | 2014 | NR | bup, iso, til, zol | mid, bup | NR | yes | ET to trach | LR 150-200 mL/hr | NR |  |
| Andresen | 2018 | ket, xyl | fen, mid | fen, ket, mid, | atra | yes | ET | NS 2- 10ml/kg/hr | NE | NC/NR |
| Mendes | 2022 | mid, ket | fen, pro | pro, fen | NR | NR | ET | NS 250 mL boluses | NE | NR |
|  |  | **Studies with Studies with PaO_2_/FiO_2_>100** | | | | |  |  |  |  |
| ***Studies that compared an MV alone group with an ECMO group*** | | | | | | | | | | |
| Zwischenberger | 1993 | Nr | halo | NR | NR | NR | trach | NR | NR | NR |
| Hayes | 2015 | NR | mid, alf | mid, alf, ket, bup | NR | Yes | ET to trach | NS, albumin | NE, epi, vaso | yes |
| MacDonald | 2015 | NR | mid, alf | mid, alf, ket, bup | NR | Yes | ET to trach | NS, albumin | NE, epi, vaso | yes |
| Du | 2016 | NR | iso | NR | NR | NR | NR | NR | NR | NR |
| Passmore | 2016 | NR | mid, alf | mid, alf, ket, bup | NR | Yes | ET to trach | NS, albumin | NE, epi, vaso | yes |
| Passmore | 2017 | NR | mid, alf | mid, alf, ket, bup | NR | Yes | ET to trach | NS, albumin | NE, epi, vaso | yes |
| Lim | 2020 | NR | zoletil, isof | NR | NR | NR | ET | NR | NR | NR |
| Stenlo | 2021 | ket, xyla | keta, mid, fen | keta, mid, fen | NR | NR | ET | LR | NE, dobu | yes |
| Kayumov | 2022 | NR | Ket, Xylazine | Isoflurane | NR | NR | ET | NR | NR | NR |
| Brusatori | 2023 | mid | azaperone, ket, sufen, pro | pro, mid, sufen | pan | NC | ET | Sterofundin ISO 2ml/hr | NE/Epi | NC |
| ***Studies that compared an MV alone group to two different ECMO groups*** | | | | | | | | | | |
| Zhang | 2021 | NR | pen | pen | pip | NR | ET | NR | NR | NR |
| ***Studies that compared differing ECMO groups*** | | | | | | | | | | |
| LeFrack#1 | 1973 | NR | pen | pen | succ | NR | ET | D5LR 70cc/hr |  |  |
| LeFrack#2 | 1973 | NR | NR | pen | succ | NR | ET | D5%, LR | NR | NR |
| Trittenwein | 1999 | ket, xyl, atro | fen, mid | ket, xyla | Pan | NR | trach | D5%, LR | NR | NR |
| Kim | 2004 | NR | NR | NR | NR | NR | NR | NR | NR | NR |
| Prat | 2015 | NR | iso | bup, mid | NR | NR | trach | NR | NR | NR |
| Xing | 2021 | NR | sumianxin, zoletil | sumianxin, zoletil | NR | NR | ET | NR | NR | NR |
| Zhang | 2022 | pen | NR | pro or sevo | NR | NR | ET | NR | NR | NR |
| ***Studies with a single group and serial measures*** | | | | | | | | | | |
| Ju | 2018 | NR | thio | thio | pan | NR | ET | BE 500 mL to 3 ml/kg/hr | NR | NR |
| Li | 2021 | NR | sev | sev | NR | NR | NR | NR | NR | NR |
| ***Studies that compared ECMO groups with and without lung injury*** | | | | | | | | | | |
| Dembinski | 2003 | aza, keta | thio | thio, fen | NR | NR | ET | HES and Crys | NR | NR |
| Shekar | 2015 | NR | alf, mid | mid, alf, ket, bup | NR | yes | ET to trach | NS, albumin | NE, epi, vaso | yes |

Alf – alfaxalone; atra – atracurium; atro – atropine; aza – azaperone; BE – balanced electrolyte; bup – buprenorphine; chlo – chlorlase; crys – crystalloid; D5LR – dextrose 5% and lactated ringers; dobu – dobutamine; epi – epinephrine; ET – endotracheal tube; fen – fentanyl; gua – guaifenesin; halo – halothane; HES – hetastarch; iso – isoflurane; ket – ketamine; LR – lactated Ringers; mid – midazolam; NC or NR – not clear or not reported; NE – norepinephrine; NS – normal saline; pan – pancuronium; pen – pentobarbital; pip – pipecironium; pro – propofol; pub – publication; remi – remifentanil; roc – roccuronium; sev – sevoflurane; suc – succinylcholine; suf – sufentanil; thio – thiopental; til – tiletamine; trach – tracheostomy; ure – urethane; vaso – vasopressin; vec – vecuronium; xyla – xlazine; zol - zolazepam
