# Supplementary material for: A systematic review of adult animal models investigating ECMO use for ARDS: where to from here
Source: Intensive Care Med Exp. 2025 Jul 18;13:74. doi: 10.1186/s40635-025-00781-5 (PMC12274183; doi:10.1186/s40635-025-00781-5)
Supplement: Supplementary file 3 — Additional file 3. [file 40635_2025_781_MOESM3_ESM.docx]

| **Sup-Table 2**. Summary of acute lung injury methods and targets in studies | | | | | | | | | | |
| --- | --- | --- | --- | --- | --- | --- | --- | --- | --- | --- |
| Author (ref) | Year | Acute Lung Injury (ALI) Methods | | | | | | | | ALI Target |
|  |  | 1st ALI | | | | 2nd ALI | | | |  |
|  |  | Agent | Dose | Route | Time | Agent/  method | Dose | Route | Time |  |
|  |  | **Studies with PaO_2_/FiO_2_≤100** | | | | | |  |  |  |
| Pilarczyk (’15) | 2015 | LL | 1000mL | IB | Q1h | NA | NA | NA | NA | PaO2 <100 |
| ***Studies that compared an MV alone group with an ECMO group*** | | | | | | | | | | |
| Plotz (’93) | 1993 | LL | 35 mL/kg x5 | IB | NR | NA | NA | NA | NA | NR |
| Germann (’96) | 1996 | OA | 0.5mL/kg | IV | 15min | NA | NA | NA | NA | Lung injury score >2.5 |
| Iglesias (’08) | 2008 | LL | 12-15mL/kg | IB | 45-60s | NA | NA | NA | NA | PaO2 /FiO2 <100 |
| Araos (’16) | 2016 | LL | NS, 30mL/kg x 4 | IB | 15min/LL | VILI | PIP=40, PEEP=0, RR=10, I:E 1:1, FiO2=1.00 | IL | 2h | PaO2/ FiO2<250 |
| Huang (’22) | 2022 | OA | 100mg/kg | IV | NR | NA | NA | NA | NA | NR |
| ***Studies that compared an MV alone group to two different ECMO groups*** | | | | | | | | | | |
| Yanos (’90) | 1990 | OA | 0.06mL/kg | IV | NR | NA | NA | NA | NA | NR |
| Johannes (’14) | 2014 | LL | 30mL/kg q10 mins | IB | NA | NA | NA | NA | NA | PaO2 = 60-80 |
| ***Studies that compared differing ECMO groups*** | | | | | | | | | | |
| Hirschl (95) | 1995 | OA | 0.07ml/kg | IV | 5min | LL | 35 cc/kg x3 | IB | 15min | AaO2 >610 torr, PaO2 <= 50 torr |
| Hirschl (’96) | 1996 | OA | 0.07ml/kg | IV | 5min | LL | 35 cc/kg x3 | IB | 15min | AaO2 >610 torr, PaO2 <= 50 torr |
| Kopp (’10) | 2010 | LL | 40mL/kg | IB | 60min | NA | NA | NA | NA | PaO2 /FiO2 <100 |
| Kopp (’12) | 2012 | LL | 40mL/kg | IB | 60min | NA | NA | NA | NA | PaO2 /FiO2 <100 |
| Araos (’19) | 2019 | LL | NS, 30mL/kg x 4 | IB | 15min/L | VILI | PIP=40, PEEP=0, RR=10, I:E 1:1, FiO2=1.00 | IL | 2h | PaO2 /FiO2 <250 |
| Dubo (’20) | 2020 | LL | NS, 30mL/kg | IB | 15min/L | VILI | PIP=40, PEEP=0, RR=10, I:E 1:1, FiO2=1.00 | IL | 2h | PaO2/ FiO2<100 |
| Millar (’20) | 2020 | OA | 0.06ml/kg | IV | 15min | LPS | 100ug | IB |  | PaO2 /FiO2 <100 mm Hg |
| Qaqish (’20) | 2020 | GJ | 4ml/kg then 2ml/kg | IB | NR | NA | NA | Na | NA | PaO2 /FiO2 <100 |
| Araos (’21) | 2021 | LL | NS, 30mL/kg x 4 | IB | 15min/L | VILI | PIP=40, PEEP=0, RR=10, I:E 1:1, FiO2=1.00 | IL | 2h | PaO2 /FiO2 <250 |
| ***Studies with a single group and serial measures*** | | | | | | | | | | |
| Booke (’95) | 1995 | VILI | PCV/PIP=35; I:E 2:1, PEEP=0 | IB | 12h | NA | NA | NA | NA | PaO2<80 on FiO2=1.0 |
| Brederlau (’06) | 2006 | LL | 30 mL/kg | IB | NR | NA | NA | NA | NA | PaO2 40-60 pm FiO2=1.0 |
| Zick (’06) | 2006 | LL | 1.5 L | IB | NA | NA | NA | NA | NA | PaO2 <100 torr on FiO2= 1.0 |
| Muellenbach (’09) | 2009 | LL | 30cc/kg q10 mins | IB | NA | NA | NA | NA | NA | PaO2< 60 mmHg on FiO2 =1.0 |
| Langer (’14) | 2014 | OA | 0.1-1.5 ml/kg | IV | NC/NR | NA | NA | NA | NA | PaO2<200 on FiO2=1.0 |
| Andresen (’18) | 2018 | LL | NS, 30ml/kg x 4 | IB | 15min/LL | VILI | PIP=40, PEEP=0, RR=10, I:E 1:1, FiO2=1.00 | IL | 2h | PaO2 /FiO2 <250 |
| Mendes (’22) | 2022 | LL | 30-40 mL/kg | IB | NA | NA | NA | NA | NA | PaO2 /FiO2 <100 |
| **Studies with Studies with PaO_2_/FiO_2_>100** | | | | | | | | | | |
| ***Studies that compared an MV alone group with an ECMO group*** | | | | | | | | | | |
| Zwischenberger (’93) | 1993 | Smoke | 10g cotton x4 | IH | NR | NA | NA | NA | NA | COHb% >80% |
| Hayes (’15) | 2015 | Smoke | 8g cotton | IH | NR | NA | NA | NA | NA | COHb% 45-50% |
| MacDonald (’15) | 2015 | Smoke | 8g cotton | IH | NR | NA | NA | NA | NA | COHb% 45-50% |
| Du (’16) | 2016 | LPS | 1mg/kg | IP | NR | NA | NA | NA | NA | NR |
| Passmore (’16) | 2016 | Smoke | 8g cotton | IH | NR | NA | NA | NA | NA | COHb% 45-50% |
| Passmore (’17) | 2017 | Smoke | 8g cotton | IH | NR | NA | NA | NA | NA | COHb% 45-50% |
| Lim (’20) | 2020 | MRSA | 75ml of 106cfu/ml | IB | NR | LPS | 1mg/kg | IV | NR | NR |
| Stenlo (’21) | 2021 | LPS | 0.33mg/kg | IB | NR | LPS | 2ug/kg/min | IV | NA | Berlin criteria |
| Kayumov (’22) | 2022 | LPS | 10mg/kg | IP | NR | NA | NA | NA | NA | NR |
| Brusatori (’23) | 2023 | OA | 0.1 mL/kg | IV | NR | NA | NA | NA | NA | PaO2 /FiO2 <150 |
|  |  | HCL | 50 mL of HCl 0.1 M | IB | NR | NA | NA | NA | NA | PaO2 /FiO2 <150 |
| ***Studies that compared an MV alone group to two different ECMO groups*** | | | | | | | | | | |
| Zhang (’21) | 2021 | OA | 0.08-0.14mg/kg | IV | NR | NA | NA | NA | NA | PaO2 /FiO2 < 200 |
| ***Studies that compared differing ECMO groups*** | | | | | | | | | | |
| LeFrack#1 (’73) | 1973 | GJ | NR | IB | NR | NA | NA | NA | NA | Qs/Qt <50% and  PaO2 = 50mmHg on FiO2= 0.4 |
| LeFrack#2 (’73) | 1973 | GJ | NR | IT | 10min | NA | NA | NA | NA | PaO2<50 on FiO2= 40%  Qs/Qt= 50% |
| Trittenwein (’99) | 1999 | LPS | 0.5mg/kg | IV | NA | HV | NA | NA | 30min |  |
| Kim (’04) | 2004 | OA | 0.1mL/kg | IV | 30min | NA | NA | NA | NA | PaO2 <60 on FiO2=0.40 PaO2 /FiO2 <150 |
| Prat (’15) | 2015 | OA | 0.1mL/kg x3 | IV | NR | NA | NA | NA | NA | PaO2 /FiO2 <200 |
| Xing (’21) | 2021 | LPS | 2mg/kg | IB | NA | LPS | 2 mg/kg | IV | NA | NR |
| Zhang (’22) | 2022 | OA | 100mg/kg | IV | NR | NA | NA | NA | NA | PaO2 /FiO2 < 300 |
| ***Studies with a single group and serial measures*** | | | | | | | | | | |
| Ju (’18) | 2018 | OA | 0.08 mL/kg | IV | NR | OA | 0.02 ml/kg | IV | Na | PaO2 /FiO2 <200 mmHg for ≥ 1h |
| Li (‘21) | 2021 | OA | 100 mg/kg | IV | NR | NA | NA | NA | NA | NA |
| ***Studies that compared ECMO groups with and without lung injury*** | | | | | | | | | | |
| Dembinski (’03) | 2003 | LL | 40 cc/kg | IB | NR | NA | NA | NA | NA | PaO2 <100 mmHg for >1hr |
| Shekar (’15) | 2015 | Smoke | 8g cotton | IH | NR | NA | NA | NA | NA | COHb% 45-50% |

AaO2 – Alveolar to arterial oxygen gradient; COHb% - carboxy hemoglobin %; cfu – colony forming units; FiO_2_ – fractional inspired oxygen concentration; HV – hypoventilation; IB – intrabronchial; I:E – ratio of inspiratory to expiratory time; IH – inhalational; IP – intraperitoneal ; IT – intratracheal; IV – intravenous; IH- Inhalation; GJ; Gastric Juice; LA – linoleic acid; LC – lung collapse; LL – lung lavage; LPS – lipopolysaccharide; NA – not applicable; NR – not reported or not clear; OA – oleic acid; PA – pulmonary artery; PaO_2_ – arterial oxygen pressure (mmHg); PCV – pressure control ventilation; PEEP – positive end expiratory pressure (cmH_2_O); PIP – peak inspiratory pressure (cmH_2_O); RA – right atrial; RR – respiratory rate (breaths per minute or targeted PaCO_2_); Rt – route; RV – right ventricular; SA – *S. aureus*; VILI – ventilator induced lung injury
