# Supplementary material for: A systematic review of adult animal models investigating ECMO use for ARDS: where to from here
Source: Intensive Care Med Exp. 2025 Jul 18;13:74. doi: 10.1186/s40635-025-00781-5 (PMC12274183; doi:10.1186/s40635-025-00781-5)
Supplement: Supplementary file 4 — Additional file 4. [file 40635_2025_781_MOESM4_ESM.docx]

| **Sup-Table 3**. ECMO methods | | | | | | | | | | |
| --- | --- | --- | --- | --- | --- | --- | --- | --- | --- | --- |
|  |  | ECMO type | | ECMO equipment | | ECMO settings | | | Anticoagulation | |
| Author (ref) | Year | Mode | Config | Pump | Oxygenator | Blood flow (BF) | Sweep gas | Membrane Gas Oxygen Fraction | Agent | ACT  goal (s) |
| **Studies with PaO_2_/FiO_2_≤100** | | | | | | | | | | |
| ***Studies that compared an MV alone group with an ECMO group*** | | | | | | | | | | |
| Plotz (’93) | 1993 | V-V | IVC-LJV | Dreissen dialysis pump | Capiox prototype | Titra to PaCO2 | NR | UC | Hep | NR |
| Germann (’96) | 1996 | V-V | IVC-SVC | Stockert Roller Pump | Maxima Plus HF | 2.5-3.5L/min | UC | 0.21-1.0 | NR | NR |
| Iglesias (’08) | 2008 | A-V | FA-FV | NovaLung | NovaLung | Intrinsic | 6-12mL/min | NR | Hep | NR |
| Araos (‘16) | 2016 | V-V | EJV-EJV | Medtronic Bioconsole 540 | HILITE 2400 LT | >65mL/kg/min | 1:1 with BF, Titra to PaCO2 | 1.0 | Hep | 180-220 |
| Huang (’22) | 2022 | V-V | RJV-RJV | PreFluid Peristaltic | Xijing | 80-90mL/min | 80-100mL/min, Titra to PaCO2 | 0.90, Titra to PaO2 | Hep | NR |
| ***Studies that compared an MV alone group to two different ECMO groups*** | | | | | | | | | | |
| Yanos (’90) | 1990 | V-V | RA-FV | BioMedicus | Sarns | NR | NR | NR | Hep | 300 |
| Johannes (’14) | 2014 | A-V | FA-FV | NovaLung | NovaLung | Intrinsic | Titra to PaCO2 | NR | Hep | NR |
| “ | 2014 | A-V | FA-FV | NovaLung | NovaLung | Intrinsic | Titra to PaCO2 | NR | Hep | NR |
| Pilarczyk (’15) | 2015 | V-V | FV-EJV | BPX80 | Maquet Quadrox D | UC | UC | NR | Hep | 180-220 |
| Pilarczyk (’15) | 2015 | V-V | FV-EJV | ILIAS | ILIAS | 2.7L/min | 3.0L/min | NR | Hep | 180-220 |
| ***Studies that compared differing ECMO groups*** | | | | | | | | | | |
| Hirschl (’95) | 1995 | V-V | RA-RIJV | NR | NR | Titra to PaO2 | Titra to PaCO2 | NR | Hep | NR |
| Hirschl (’96) | 1996 | V-V | RA-RIJV | NR | NR | Titra to PaO2 | Titra to PaCO2 | NR | Hep | NR |
| Kopp (’10) | 2010 | V-V | FV-EVJ | Bio-Pump | Affinity PRF | 1-2L/min | 3L/min | UC | Hep | 120-150 |
| Kopp (’10) | 2010 | Mini-V-V | FV-EVJ | Delta-stream | HILITE 7000 | 1-2L/min | 3L/MIN | UC | Hep | 120-150 |
| Kopp (’10) | 2010 | A-V | FV-FA | NovaLung | NovaLung | Intrinsic | 6L/MIN | UC | Hep | 120-150 |
| Kopp (’12) | 2012 | Mini-V-V | FV-EVJ | Delta-stream | HILITE 7000 | 1-2L/min | 3L/MIN | UC | Hep | 120-150 |
| Kopp (’12) | 2012 | A-V | FV-FA | Novalung | Novalung | Intrinsic | 6L/MIN | UC | Hep | 120-150 |
| Araos (‘19) | 2019 | V-V | EJV-EJV | Medtronic Bioconsole 540 | HILITE 2400 LT | >60mL/kg/min | 1:1 with BF, Titra to PaCO2 | 1.0 | Hep | 180-220 |
| “ | 2019 | V-V | EJV-EJV | Medtronic Bioconsole 540 | HILITE 2400 LT | >60mL/kg/min | 1:1 with BF, Titra to PaCO2 | 1.0 | Hep | 180-220 |
| Dubo (’20) | 2020 | V-V | RJV-RJV | Medtronic BioMedicus 540 | HILITE 2400LT | 60-70ml/kg/min | Titra to PaCO2 | 1.0 | Hep | 180-220 |
| Millar (’20) | 2020 | V-V | IVC-RA | RotaFlow | Quadrox PLS | 2/3 CO | 80% of BF, Titra to PaCO2 | 1.0 | Hep | 180-210 |
| Qaqish (’20) | 2020 | V-V | FV-EJV | Sorin Centrifugal pump | Quadreox-I Adult | Titra to MAP | 2L/min | NR | Hep | NR |
| Araos (‘21) | 2021 | V-V | EJV-EJV | Medtronic Bioconsole 540 | HILITE 2400 LT | >60mL/kg/min | 1:1 with BF, Titra to PaCO2 | 1.0 | Hep | 180-220 |
| ***Studies with a single group and serial measures*** | | | | | | | | | | |
| Booke (’95) | 1995 | V-V | RJV-RJV | Bio-Medicus | Sorin Biomedica OXY 41 | Varied | NR | NR | Hep | 150-189 |
| Brederlau (’06) | 2006 | A-V | FA-FA | NovaLung | NovaLung | Intrinsic | Varied | 1.0 | Hep | 200-250 |
| Zick (’06) | 2006 | A-V | FA-R/LFV | NovaLung | NovaLung | Intrinsic | 0-2L/min | NR | Hep | NR |
| Muellenbach (’09) | 2009 | A-V | FA-FV | NovaLung | NovaLung | Intrinsic | 10L/min | UC | Hep | 300-400 |
| Langer (’14) | 2014 | V-V | EJV-EJV | Maquet Cardiohelp | Maquet HLS Set | 2L/min | 1-10L/min | 0.5 | Hep | >160 |
| Andresen (’18) | 2018 | V-V | EJV-EJV | Medtronic Biomedicus 540 | HILITE 2400 LT | NR | NR | NR | NR | NR |
| Mendes (’22) | 2022 | V-V | RA-FV | NR | Biocube 4000 | 80mL/kg | Varied | NR | Hep | NR |
| **Studies with Studies with PaO_2_/FiO_2_>100** | | | | | | | | | | |
| ***Studies that compared an MV alone group with an ECMO group*** | | | | | | | | | | |
| Zwischenberger (’93) | 1993 | V-V/V-V | IJ-CA/FV | Roller pump | SciMed | 39/mL/kg/min | 10L/min, Titra to PaCO2 | NR | Hep | 200-300 |
| Hayes (’15) | 2015 | V-V | JV-JV | Medtronic Bioconsole 550 | Quadrox | 66% of CO | Titra to PaCO2 | 1.0 | Hep | 200-300 |
| MacDonald (’15) | 2015 | V-V | IVC-RA | RotaFlow | Quadrox PLS | 66% of CO | 0.8:1 of BF | 1.0 | Hep | 200-300 |
| Du (’16) | 2016 | V-V | NA | NR | NR | NR | NR | NR | NR | NR |
| Passmore (’16) | 2016 | V-V | IVC-RA | RotaFlow | PLS Quadrox D | 2/3 | 80% of BF | 1.0 | Hep | 200-300 |
| Passmore (’17) | 2017 | V-V | IVC-RA | RotaFlow | PLS Quadrox D | 2/3 | 80% of BF | 1.0 | Hep | 200-300 |
| Lim (’20) | 2020 | V-V | RFV-RFV | RotaFlow | Quadrox PLS | 50mL/kg/min | 1:1 with BF, Titra to PaCO2 | 1.0 | NR | NR |
| Stenlo (’21) | 2021 | V-V | RA-AO | Affinity CP | Affinity Fusion | 75% of CO | NR | NR | Hep | 180-220 |
| Kayumov (’22) | 2022 | V-V | REJV-LFA | Watson-Marlow Pumps | Micro-1 | 50ml/min | NR | NR | Hep | NR |
| Brusatori (’23) | 2023 | V-V | EJV-RFV | Rotaflow (Getinge) | Quadrox PLS | 50-60ml/kg/min | 1:1 with BF | NR | Hep | >250 |
| ***Studies that compared an MV alone group to two different ECMO groups*** | | | | | | | | | | |
| Zhang (’21) | 2021 | V-V | RFV-LIJ | Roller-pump | NR | 350-400mL/min | 8L/min | 1.0 | Hep | 200-250 |
| ***Studies that compared differing ECMO groups*** | | | | | | | | | | |
| Lefrack #1 | 1973 | V-V | IVC-SVC | Roller Pump | Edwards 3m2 | Varied | NR | UC | NR | NR |
| Lefrack #2 | 1973 | V-V | IVC-SVC | Roller Pump | Travenol 1.5m2 | Varied | NR | UC | NR | NR |
| Lefrack #2 | 1973 | V-V | IVC-SVC | Roller Pump | Pierce-GE 2m2 | Varied | NR | UC | NR | NR |
| Lefrack #2 | 1973 | V-V | IVC-SVC | Roller Pump | Lande-Edwards 3m2 | Varied | NR | UC | NR | NR |
| Trittenwein (’99) | 1999 | V-V | RJV-RCA | RS 7800 Minipump | Jostra M5 | 80mL/kg/min | NR | 1.0 | Hep | NR |
| Trittenwein (’99) | 1999 | V-V | RJV-LSV | RS 7800 Minipump | Jostra M5 | 80mL/kg/min | NR | 1.0 | Hep | NR |
| Kim (’04) | 2004 | V-V | RA-Ao | Nonpulsatile BPX80 | Capiox SX10 | 1.8-2L/min | 1:1 with BF | 0.60 | NR | NR |
| Kim (’04) | 2004 | V-V | RA-Ao | Pulsatile Prototype | Capiox SX10 | 1.8-2L/min | 1:1 with BF | 0.60 | NR | NR |
| Kim (’04) | 2004 | V-V | RA-Ao | Pulsatile Prototype/CC | Capiox SX10 | 1.8-2L/min | 1:1 with BF | 0.60 | NR | NR |
| Prat (’15) | 2015 | V-V | IVC-RA | CardioHelp | CardioHelp | 2L/min | NR | NR | Hep | 160-180# |
| Xing (’21) | 2021 | V-V | RIJ-RCA | Kewei | Kewei | 150mL/min | 200mL/min | 1.0 | NR | NR |
| Zhang (’22) | 2022 | V-V | RJV-RJV | PreFluid Peristaltic | Xijing | 80-90mL/min | 80-100mL/min | 0.90 | Hep | NR |
| ***Studies with a single group and serial measures*** | | | | | | | | | | |
| Ju (’18) | 2018 | A-V | FA-IJV | NovaLung | NovaLung | Intrinsic | 3 x BF | 1.0 | Hep | 120-160 |
| Li (’21) | 2021 | V-V | RJV-RJV | PreFluid Peristaltic | Xijing | 80-90mL/min | 80-100mL/min, Titra to PaCO2 | 0.90, Titra to PaO2 | Hep | NR |
| ***Studies that compared ECMO groups with and without lung injury*** | | | | | | | | | | |
| Dembinski (’03) | 2003 | V-V | FV-FV | Delta Stream | HILITE 7000 | 30% of CO | 1:1 with BF | 1.0 | Hep | 130s |
| Shekar (’15) | 2015 | V-V | IVC-FV | Maquet Rotaflow | Maquet Quadrex D | 60-80mL/kg/min | 80% of BF | 1.0 | Hep | 220-250 |

AO – aorta; A-V – arteriovenous; BF – blood flow; BPX80 – Biomedicus Centrifugal (Medtronix); CC – compliance chamber; CO – cardiac output; Config. – configuration; CPAP – continuous positive airway pressure; ECLA – extracorporeal lung assist; vol – volume; Hep – heparin; HF – hollow fibert; ILIAS – miniaturized system with a blood pump within an oxygenator; Maquet Adult Quadrox D; MAP – mean arterial blood pressure; NA – not available; PCV – pressure controlled ventilation; Titra – titrated to an arterial partial oxygen pressure target; UC – unclear; Titra – titrated; VCV – volume controlled ventilation; V-V – venovenous;

*Ventilation with ECMO; **Varied – indicates the parameter was varied based on study group or protocol; ^#^ only one group was monitored
